# Theory and experimental verification of configurable computing with stochastic memristors

**DOI:** 10.1038/s41598-021-83382-y

**Published:** 2021-02-18

**Authors:** Rawan Naous, Anne Siemon, Michael Schulten, Hamzah Alahmadi, Andreas Kindsmüller, Michael Lübben, Arne Heittmann, Rainer Waser, Khaled Nabil Salama, Stephan Menzel

**Affiliations:** 1 Department of Electrical Engineering and Computer Science, University of California Berkeley, Berkeley, CA USA; 2 Computer, Electrical and Mathematical Sciences and Engineering Division, King Abdullah University of Science and Technology, Thuwal, Saudi Arabia; 3 Institut für Werkstoffe der Elektrotechnik II (IWE II), RWTH Aachen University, Aachen, Germany; 4 JARA - Fundamentals for Future Information Technology, Jülich, Germany; 5 Peter Grünberg Institut 10 (PGI-10), Forschungszentrum Jülich GmbH, Jülich, Germany; 6 Peter Grünberg Institut 7 (PGI-7), Forschungszentrum Jülich GmbH, Jülich, Germany

**Keywords:** Electronic devices, Electrical and electronic engineering, Information technology

## Abstract

The inevitable variability within electronic devices causes strict constraints on operation, reliability and scalability of the circuit design. However, when a compromise arises among the different performance metrics, area, time and energy, variability then loosens the tight requirements and allows for further savings in an alternative design scope. To that end, unconventional computing approaches are revived in the form of approximate computing, particularly tuned for resource-constrained mobile computing. In this paper, a proof-of-concept of the approximate computing paradigm using memristors is demonstrated. Stochastic memristors are used as the main building block of probabilistic logic gates. As will be shown in this paper, the stochasticity of memristors’ switching characteristics is tightly bound to the supply voltage and hence to power consumption. By scaling of the supply voltage to appropriate levels stochasticity gets increased. In order to guide the design process of approximate circuits based on memristors a realistic device model needs to be elaborated with explicit emphasis of the probabilistic switching behavior. Theoretical formulation, probabilistic analysis, and simulation of the underlying logic circuits and operations are introduced. Moreover, the expected output behavior is verified with the experimental measurements of valence change memory cells. Hence, it is shown how the precision of the output is varied for the sake of the attainable gains at different levels of available design metrics. This approach represents the first proposition along with physical verification and mapping to real devices that combines stochastic memristors into unconventional computing approaches.

## Introduction

The wide spread deployment of smart and cognitive devices along with the amount of communication data are enforcing high levels of computational and operational requirements. On the other hand, the underlying technological advancements are lagging behind in this fast pace of Big Data era. Hence, to cope with the current demands and allow for further innovations, a radical change in the design of computing systems at all stack levels needs to be adopted^1–4^. Innovations in the architectures and devices are to be integrated together to reach the performance required for the current applications. From an architectural aspect, one of the main alternatives is shifting from conventional Von-Neumann systems towards Processing-in-memory^5^. The concept of computational memory has shown to provide high energy efficiency with the memory elements used for storage along with processing tasks. This operation minimizes the costly communication bottleneck with the memory to fetch the elements for the logic operations. Further to these operational demands, having non-volatile memories with fast write/access speed is desirable.

Bipolar resistive switching devices (BRS), a subclass of memristive devices or short memristors, are potential candidates for the current computationally and memory demanding applications. They offer fast write/read speed, non-volatility and high scaling potential and can be also used as basic elements in logic-in-memory concepts. In BRS devices the binary information is encoded by different resistance states, namely the low resistive state (LRS) and the high resistive state (HRS). To switch between the LRS and the HRS appropriate voltage stimuli need to be applied. In bipolar switching devices one voltage polarity is required to switch the device from the HRS to the LRS, i.e. the SET process, whereas the opposite voltage polarity is required to reset the cell from the LRS to the HRS, i.e. the RESET process^6–8^. Several approaches have been proposed for the use of bipolar resistive switching devices for logic circuits. One of the first approaches, ‘IMPLY’-logic, assumed the imply operator as the base for the Boolean gates^9^. More complex functionality like an adder are also shown by means of simulation for this approach^10,11^. Another approach suggests a CMOS-like sequential logic. Here traditional logic gates are mapped one by one to the memristive logic gates^12^. The BRS devices need to be programmed for each input signal, so that the output voltage corresponds to the result of the function. A third logic approach is the so-called ‘CRS-logic’ (Complementary Resistive Switch-logic), which is a sequential logic approach, too. It can perform 14 out of 16 two-input Boolean operations with just one device^13,14^. For this approach, also more complex functions have been experimentally demonstrated, like adders^15^. Since the IMPLY-logic and the CRS-logic are designed to work in BRS crossbar arrays, they pave the way for in-memory computing and further innovations as well. On top of computational innovations, a wide range of current applications exhibit high tolerance to errors, which in turn reflects on a looser accuracy requirement for the underlying hardware. These compromises pave the way for the reviving of approximate computing approach.

In this work, we investigate the feasibility of approximate computing concepts using memristive elements. The intrinsic stochasticity of the switching process is exploited to implement CRS-logic gates with adjustable level of reliability. Using a mathematical model for the switching variability and the CRS-logic approach, we establish theoretical formulations and limits to the consequent impact of variability on standalone gates. Moreover, the basics of the concept of reliable computing with unreliable components is verified experimentally using bipolar resistive switching devices. Almost exact mapping is attained between the theoretical and physical measurements. The devices show the required switching variability and the functionality of a NAND gate with adjustable level of reliability is demonstrated. It is found that the experimental data deviates slightly from the idealized theoretical formulation. The cause of this deviation is discussed with respect to the device physics. Based on these results device requirements for approximate computing applications are deduced.

## Results

### Probabilistic memristive logic

In this work, the CRS-logic approach is chosen to implement a probabilistic memristive logic^2,16^. The CRS-logic relies on using the two terminals ($T_1$ and $T_2$) of a bipolar resistive switching element as the inputs of any logic operation (cf. Fig. 2a). The final result is then stored directly in the BRS device. The logic states ‘0’ and ‘1’ are represented by a high and a low potential, respectively. In contrast, the result is encoded as the resistance states of the BRS device; the HRS and LRS resemble logic ‘0’ and ‘1’, respectively. The BRS device is assumed to switch to the LRS or logic ‘1’  if $T_1$ is at high potential (logic ‘1’) and $T_2$ is at low potential (logic ‘0’). For a low potential at $T_1$ and a high potential at $T_2$ the BRS devices switches back to the HRS or the logic state ‘0’ . The state does not change when both terminals are at the same potential, as the voltage drop would be zero. Thus, the device switches only for specific combination of inputs. In order to realize a Boolean operation, a sequence of cycles needs to be applied. The first cycle is considered as the initialization stage, where the BRS device is put in a predetermined state (LRS or HRS). This state depends on the implemented logic gate. For implementing the gates AND, OR and IMP, for example, the memristive device is initialized to the LRS, whereas it is initialized to the HRS for the NOT or RNIMP gate, for example. This step is followed by one or two logic steps depending on the selected logic function^13,14^. The final result is stored in the cell’s resistance.

To implement a probabilistic memristive logic approach, a set of assumptions are put into this analysis to ensure a common ground and to provide a simple overview of the expected outcome.

1. The initialization stage is deterministic. To this end, a stronger SET is required as in the succeeding steps.
2. The inputs p and q to the logic gates are deterministic with uniform distribution.
3. The operation of the memristor is probabilistic with a generic switching probability $P_\text {s}(V, \Delta t_\text {p})$ that is governed by the characteristic of the device. It depends on the applied voltage *V* and the length $\Delta t_\text {p}$ of the voltage pulse.
4. SET and RESET probability are assumed to be equal. To this end, |*V*| and $\Delta t_\text {p}$ need to be chosen appropriately.

**Figure 1:**
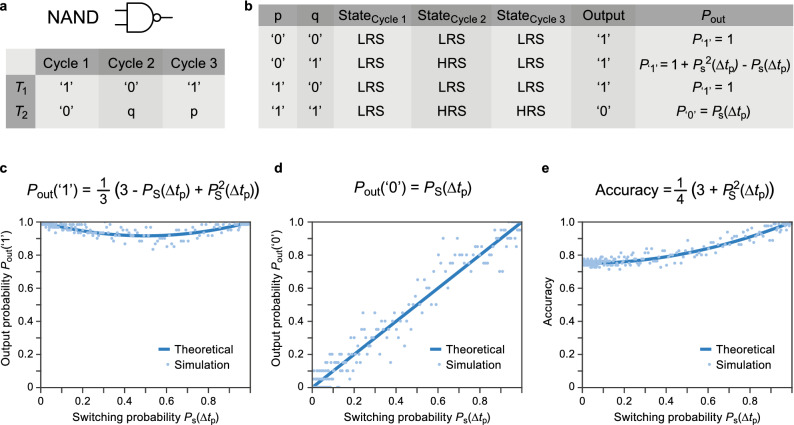
(**a**) The input scheme, which needs to be applied to receive a NAND functionality. (**b**) The truth table for the NAND gate with the added probabilistic output behavior for each entry. (**c**) The output probabilities for having a correct ‘1’ on average for all the entries. (**d**) The output probabilities for having a correct ‘0’ at the output of the gate. (**e**) The accuracy of the output bit on average with respect to the switching probability of the BRS device.

Figure 1a shows the implementation of a NAND gate in CRS logic. The first cycle is the initialization cycle, which is deterministic according to our definitions. To this end logic ‘1’ is applied to $T_1$ and ‘0’ is applied to $T_2$, resulting in a positive voltage drop over the device, which sets the device to the LRS. Thus, the device will be always in the LRS after cycle 1 (cf. $\hbox {State}_\text {Cycle,1}$ in Fig. 1b). In the second cycle, a ‘0’  is applied to $T_1$, while q is applied to $T_2$. If q is ‘0’ , there is no voltage drop over the device and it remains in the LRS. In contrast, q = ‘1’  leads to a negative voltage drop and the device switches to the HRS as shown in the column $\hbox {State}_\text {Cycle,2}$. In the last cycle, a ‘1’  is applied to $T_1$, while p is applied to $T_2$. Thus, only if p is ‘0’ a voltage is applied to the device and the device resistance can be changed to the LRS. The final state and the corresponding logic output after this final cycle is shown in the column $\hbox {State}_\text {Cycle,3}$ and Output of Fig 1b. When comparing the input combinations p, q with the output, it becomes clear that this procedure implements a NAND gate. With this procedure 14 out of 16 Boolean logic functions can be implemented^13,14^. Further gates and the corresponding truth tables are presented in the supplementary information. The CRS logic relies on the conditional switching of the memristive device, which is ideally deterministic. By reducing the applied voltage or reducing the pulse length, however, the switching becomes probabilistic. For each entry of the truth table in Fig 1b, the certainty of having a correct output, whether it is ‘0’ or ‘1’, is characterized by the output probability $P_\text {out}$. The latter is determined by the switching probability of the BRS device at a particular voltage and time $\Delta t_\text {p}$ and the sequence of the applied input signals. Four different combinations of the input pair (p, q) are available as shown in Fig. 1b.

For the logical input combination (p, q) = (‘0’, ‘0’) the second cycle will not have any effect on the device state as $T_1$ and $T_2$ are set to the same potential and thus there is no voltage drop across the device. For the third cycle, a positive voltage is applied, which will also not change the state as the device is already in the LRS. Thus, the (‘0’, ‘0’) case will always produce a correct output ‘1’ . This means that the probability to receive a ‘1’  as the output is 100% $(P_{\text {(`0',`0')}}\text {(`1')} = 1)$ and the probability is $P_{\text {(`0',`0')}}(\text {`0'}) = 0$ to receive a ‘0’. For the pair (p, q) = (‘0’, ‘1’), the correct output ‘1’  is received if the BRS devices would switch in both cycles (in the 2nd to the HRS and in the 3rd to the LRS) or if it would not switch in the second cycle. In the latter case, the BRS device is still in the LRS and in the third cycle a SET pulse is applied, which does not change the result. The probability of getting the correct output is then

1 $$\begin{aligned} P_{(\text {`0',`1')}} (\text {` 1'})=P_\text {s}^2 (\Delta t_\text {p})+(1-P_\text {s} (\Delta t_\text {p}))\cdot 1. \end{aligned}$$

For the pair (p, q) = (‘1’, ‘0’), there is no voltage drop over the device in the second and third cycle. Thus, the initial state cannot be changed and is directly the output, so $P_{\text {(`1', `0')}}\text {(`1')} = 1$.

For the pair (‘1’, ‘1’), only the second cycle determines whether the output is correct or not as there is no voltage drop over the device in the third cycle. Thus, the probability of achieving the correct output ‘0’   is determined by the switching probability to the HRS of the BRS device in the second cycle; i.e. the probability $P_{\text {(`1', `1')}}\text {(`0')} = P_\text {s}(\Delta t_\text {p})$. The overall probabilities of the NAND gate to obtain the correct outputs ‘0’   and ‘1’   are then

2 $$\begin{aligned} P_\text {out}\text {(`0')} = P_\text {s}(\Delta t_\text {p}) \end{aligned}$$

and

3 $$\begin{aligned} P_\text {out}\text {(`1')}= \frac{1}{3}(3-P_\text {s}(\Delta t_\text {p})+P_\text {s}^2(\Delta t_\text {p})). \end{aligned}$$

In addition to the single bit probabilities, the behavior of the gate is further quantified with the notion of accuracy. It is specified as

4 $$\begin{aligned} \text {Accuracy}= \frac{N_\text {`0'}}{N}P_\text {out}\text {(`0')}+ \frac{N_\text {`1'}}{N}P_\text {out}\text {(`1')}\, \end{aligned}$$

where *N* corresponds to the total number of entries in a particular truth table. $N_\text {`1'}$ is the number of entries with the output set to ‘1’, and $N_\text {`0'}$ is the number of entries with the output set to ‘0’. The accuracy for the NAND operator would thus be calculated as follows

5 $$\begin{aligned} \text {Accuracy}_\text {NAND}= \frac{1}{4}P_\text {s}(\Delta t_\text {p})+ \frac{3}{4}(\frac{1}{3}(3-P_\text {s}(\Delta t_\text {p})+P_\text {s}^2(\Delta t_\text {p})))=\frac{1}{4} (3+P_\text {s}^2(\Delta t_\text {p})). \end{aligned}$$

Figure 1c–e show the output probabilities for $P_\text {out}$(‘1’), the output probabilities for $P_\text {out}$(‘0’), and the NAND gate accuracy for different assumed switching probabilities, respectively. The solid line represents the theoretical probabilistic analysis and the blue dots the simulation data obtained by using the stochastic model^17^, which is outlined briefly in the methods section. The NAND gate shows a high accuracy starting from 0.75 since the deterministic initialization state does not need to be changed in three of the possible gate input combinations. Hence, a large space for saving energy or time is feasible for simple logic operations by scaling the voltage or the cycle time. A similar analysis is conducted for the remaining gates that require one to three cycles for operation. The analyses are shown in the supplement (Figs. S7–S11).

### Experimental

**Figure 2:**
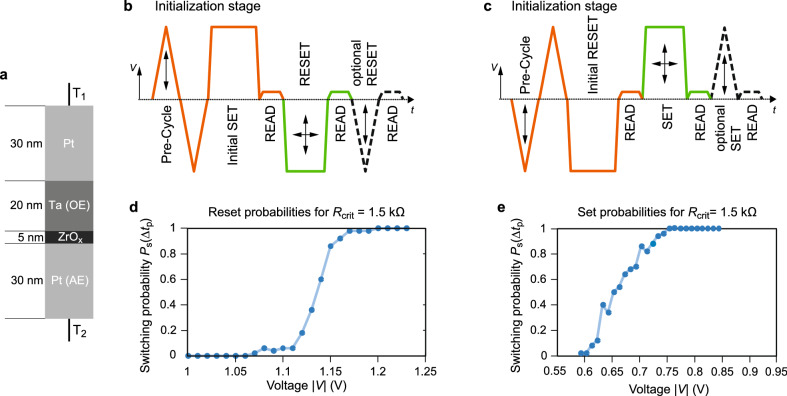
Measurements for SET and RESET probabilities using $\hbox {ZrO}_x$-based VCM devices. (**a**) Device stack. (**b**,**c**) Pulse scheme to measure the RESET (SET) switching probabilities. The potential is applied to $T_1$ while $T_2$ is grounded. In the initialization stage (orange), first a pre-cycle consisting of a 2.76 ms (3.31 ms) long positive (negative) triangular voltage pulse with variable amplitude starting with 1 V (− 1.2 V) and a 3.31 ms (2.76 ms) long negative (positive) triangular voltage sweep with a peak voltage of − 1.2 V (1 V) is applied once to unstuck the device. Then, an initial 10 $\mu$s long SET (RESET) pulse of 1 V (− 1.2 V) is used to set the wanted device state. Afterwards, the device state is verified by a 0.1 V READ pulse of 1 ms length to secure the uniformity of the initial states. If the read state differs too much from the targeted initial state, these pulses are repeated. For each repetition the peak voltage of the pre-cycle is increased (decreased) by 0.05 V. After this initialization phase, the actual SET or RESET pulses are conducted (green). The 10 $\mu$s rectangular pulses were varied in their height in order to achieve different switching probabilities. The outcome is checked by a 0.1 V READ pulse of 1 ms length. If the device was switched, the next cycle begins. Otherwise an additional optional 1.66 ms long SET (RESET) triangular-pulse is applied and the state is verified by a subsequent 1 ms long, 0.1 V READ pulse. If the switching after the additional pulse was still not successful, i.e. the read current is > 50 $\mu$A ($<\,130\, \mu$A), the optional pulse is repeated with a higher absolute peak voltage (+0.05 V). (**d**,**e**) RESET/SET switching probabilities of 50 cycles.

To verify the theory, electrical measurements are performed. The most promising BRS devices rely on the electrochemical metallization mechanism (ECM) or the valence change mechanism (VCM)^6^, since both devices can offer a very high device density. The devices can be implemented in architectures offering 4*F*$^2$ cells and are 3D stackable^18^. Due to the simple metal insulator metal structure the fabrication cost of these devices is expected to be very low^19^, but since the technology is still an emerging technology there is no reliable source for actual data. To cover both type of devices a $\hbox {ZrO}_x$-based VCM device and a $\hbox {SiO}_2$/CuS/Cu-based ECM device were tested. The information of the fabrication procedure for both types can be found in the Method section. In a Cu-based ECM cell, a filament consisting of the metal atoms of the active Cu electrode is formed/ dissolved to switch between the LRS and the HRS^20^. In a filamentary-switching VCM device the switching is based on the reconfiguration of oxygen defects within a filamentary region^6^. Hence, ECM as well as VCM devices are changing their atomic configuration to switch between the two states. Due to this nature, both type of devices have a high variability in the switching process and in the stored states. Thus, both should meet the requirement of probabilistic switching.

First, the assumption of probabilistic switching needs to be confirmed for the devices under test. For this, the switching probabilities of SET and RESET are measured by applying a strong initial pulse followed by a pulse, which can vary in height and width. The initial pulse needs to be strong enough to secure the same initial state of the operation without any influence of the former state. Figure 2a depicts the device stack of the used $\hbox {ZrO}_x$-based VCM device. A strong ‘ sticking’ effect was found in these devices. This means the device cannot be switched successfully to the opposite state unless a higher voltage or longer time is used. The device ‘ sticks’  to its state. This temporary sticking effect can be attributed to the state-dependence of the switching process as outlined in the discussion part. Apart from this temporary sticking effect, sticking effects can also terminate the device lifetime. In literature, stuck-in-LRS^21–23^, stuck-in-HRS^21,22^, and even stuck-in-intermediate-state^24^ errors have been reported. A possible origin for these failures is the loss of oxygen defects to the electrodes or the surrounding. In addition, cycling can lead to phase segregations or metallic enrichments^25,26^ or in-diffusion of metal particles from the electrodes^27^. In our experiments, a temporary sticking effect appears if the cell is switched consecutively with the same polarity for several times. Due to this effect, the success rate showed a strong dependence on the device history. Since the sticking effect of the fabricated devices is problematic to the application, refresh cycles were introduced. The resulting pulse schemes are depicted in Fig. 2b,c. The pulse sequence has three stages: an initialization stage (orange), the actual SET/RESET sequence (green) stage and an optional ‘ unsticking’  stage. The optional stage is carried out if the device did not switch. The state, and thus the switching, is defined as LRS (HRS) if the resistance is lower (higher) than a critical resistance $R_\text {crit}$ = 1.5 k$\Omega$ in the read step. This pulse scheme is repeated ten times with the same pulse height. After that the pulse height is increased until the maximum voltage is reached. This is repeated five times, thus, all voltages are measured 50 times. The measurement results show a probabilistic dependence of the switching process on the applied voltage as depicted in Fig. 2d,e. Similar results could be achieved for an ECM device (Figs. S3 and S4) and without the unsticking preventions for the same VCM device (Fig. S1). Based on these results a certain switching probability can be achieved for 10 µs long pulses by choosing an appropriate SET and RESET voltage, e.g. there is a 0.92 chance that a RESET occurs in 10 µs if the voltage is chosen to be 1.16 V.

**Figure 3:**
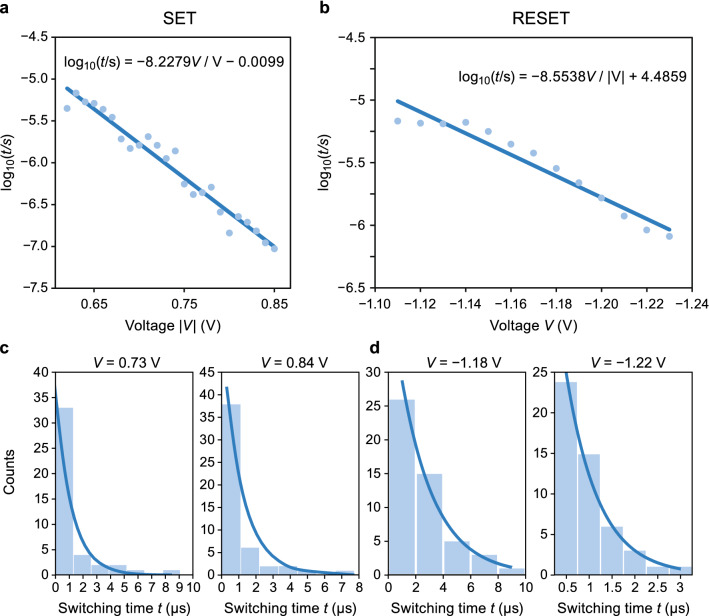
The time-voltage relationship for the VCM device during the (**a**) SET operation and (**b**) RESET operation. The experimental fitting for the switching times at different voltages to the exponential distribution, leading to a Poisson process for the switching events (**c**) for the SET process and (**d**) the RESET process. The blue line in (**c**) and (**d**) is calculated assuming a Poisson distribution function that is scaled to the number of cycles. The fit equation reads $f = 1.1\cdot h_\text {max} \cdot \text {exp}(-(t-t_\text {min})/\tau )$ where $h_\text {max}$ is equal to the number of counts in the first bin, $t_\text {min}$ is the minimum switching time measured and $\tau$ is the mean switching time equal to the blue dots in (**a**) and (**b**).

As the current transients are recorded during the pulse experiment, the exact switching time can be extracted by defining a critical current level (cf. Fig. S5). The recorded mean switching times of the 50 cycles show an exponential dependence on the applied voltage *V* as illustrated in Fig. 3a for the SET and in Fig. 3b for the RESET. This behavior shows the highly nonlinear switching kinetics, which is typically observed in ECM and VCM devices^28^ . The relation between the switching time and voltage can be approximated by

6 $$\begin{aligned} \text {log}_{10}(\tau )=\alpha _\text {(SET/RESET)} V+\epsilon _\text {(SET/RESET)} \end{aligned}$$

where $\alpha _\text {(SET/RESET)}$ and $\epsilon _\text {(SET/RESET)}$ are fitting parameters that are extracted from the measurements. The resulting values are given as inset in Fig. 3a,b. These parameters are used as input for the stochastic model outlined in the Methods section. To further quantify the statistical behavior of the device in response to the applied voltage, the mean switching times are analyzed. The distribution of the switching times at a particular voltage has an exponential dependency of the time^17,29^, which is illustrated in the histograms of Fig. 3c,d. Thus, the switching events follow a Poisson process and the switching probability $P_\text {s}(\Delta t_\text {p})$ is given by

7 $$\begin{aligned} P_\text {s}(\Delta t_\text {p})=1-e^{(-\Delta t_\text {p}/\tau (V))}. \end{aligned}$$

Based on these results, a certain switching probability is also achieved by keeping the voltage constant and varying the switching time. In literature, it was shown that other device stacks show a log-normal distribution of the switching times^30^ instead of the Poisson distribution here. In this case, the probability is a function of the applied voltage (and pulse widths), too. Thus, the approximate computing concept discussed in this paper can be performed with such devices as well (cf.^17^).

**Figure 4:**
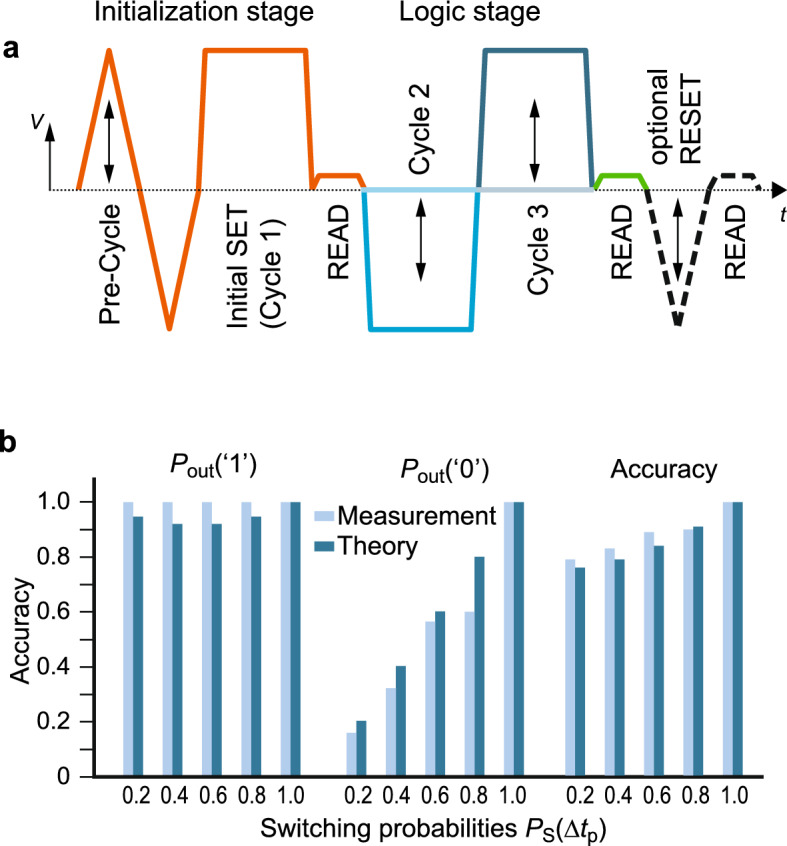
NAND gate measurements. (**a**) Pulse scheme for the NAND gate measurements. In the initialization stage (orange), first a pre-cycle consisting of a 3.31 ms long positive (negative) triangular voltage pulse with variable amplitude starting with 1 V and a 3.31 ms long negative triangular voltage sweep with a peak voltage of -1.2 V is applied once to unstuck the device. Then, an initial 10 $\mu$s long SET pulse (Cycle 1) of 1 V is used to set the wanted device state. Afterwards, the device state is verified by a 0.1 V READ pulse of 1 ms length to secure the uniformity of the initial states. If the read state differs too much from the targeted initial state,i.e. $I_\text {read} < 130\,\mu A$, these pulses are repeated. For each repetition the peak voltage of the pre-cycle is increased by 0.05 V. After this initialization phase, the Logic stage consists of two successive 10 $\mu$s long rectangular pulses. The amplitudes depend on the chosen switching probability and the logic inputs. The outcome is checked by a 0.1 V READ pulse of 1 ms length. If the read current is larger than 50 $\mu$s, an optional 3.31 ms long RESET triangular-pulse of − 1.2 V is applied and the state is verified by a subsequent 1 ms long, 0.1 V READ pulse. If the switching after the additional pulse was still not successful, i.e. the read current is > 50 $\mu$A , the optional pulse is repeated with a higher absolute peak voltage (+0.05 V). (**b**) Comparison of the theoretical (dark blue) and measurement results (light blue) of the NAND gate using the 0.2, 0.4, 0.6, 0.8 and 1.0 points of the SET/RESET switching probabilities.

To study the feasibility of logic cascades, a NAND gate with two consecutive logic cycles is measured using the 0.2 to 1.0 switching probability voltages extracted from the measurements shown in Fig. 2d,e, respectively. The NAND gate involves the switching to the HRS and the switching back to the LRS in cycles 2 and 3. Thus, it can be regarded as representative for the other gates. If the concept works for the NAND gate, it should work for the other gates as well. In the experiment, a constant pulse length of 10 µs is applied for SET and RESET. As before, to avoid the sticking effect the pulse scheme starts with a full switching cycle followed by an initialization pulse to set the device in the LRS. After a READ pulse, the two logic pulses are applied depending on the inputs p and q. After checking the outcome by a READ pulse an optional RESET can be performed (Fig. 4a).

In Fig. 4b the measured output probabilities of the NAND gate are depicted. The probability of ‘0’  and the accuracy of the whole gate show the same trend as expected from the theory, whereas the probability of ‘1’ deviates slightly from the theory. If the probabilities are displayed for each input pair (supplement Fig. S6) only the combination p = ‘0’ and q = ‘1’ shows a different trend as expected from the theory. The reason for this lies in the dependence of the switching speed on the former device state^31^, i.e., the internal atomic configuration. For this input combination the first logic step should be a RESET step. Since the RESET pulse has a shorter pulse width and a reduced height compared to the initial RESET pulse of the SET switching probability measurements, the device state is most likely in a less resistive state than in the SET probability measurements. This results in an easier succeeding SET process. Thus, the SET switching probability would be higher and therefore the outcome of this combination is in this case better than expected by the theory.

## Discussion

Based on these measurements, device requirements can be derived for this application. To keep approximately the same switching probability, the state-dependence of the switching should be as small as possible. The state-dependence can lead to two problems. The first one is the sticking effect. If the device is switched to a very high resistive state, a higher voltage is required to switch back to the LRS. Based on the switching mechanism this problem is more pronounced for VCM devices as the switching process is thermally-assisted via Joule heating^32^. In ECM devices, in contrast, the SET process is mainly electric field accelerated^33,34^, since the power dissipation is too small to result in relevant Joule heating during the switching. Thus, the effect should be smaller. ECM and VCM devices suffer similarly from sticking to the LRS as it is connected to an internal series resistance. If the device is programmed to a very low ohmic state, a large portion of the applied voltage will drop over the series resistance and is thus not available to drive the RESET transition. The RESET voltage will increase for less resistive LRS^35^. To avoid the sticking problem, a device or a circuit would be needed, which prevents the device from getting too low ohmic or too high ohmic. The second problem that arises from the state-dependence of the switching occurs if the device assumes an intermediate state close to the critical resistance. In this case the SET or RESET pulse will switch the device easily with a higher probability than it is supposed to. The pulse might even program it to a very low resistive (very high resistive) state causing a sticking problem for the subsequent cycle. This problem can only be solved if the device switches more abrupt between LRS or HRS rather than gradual, which facilitates the switching to intermediate resistance states.

A second device requirement for sequential logic is a high switching endurance. The necessity of additional refresh cycles, would pose an even higher constraint. Until now $10^{12}$ cycles for single VCM devices^36^ and $10^{10}$ for single ECM devices^37^ could be shown, which could be improved in future technologies. Another possibility is an active device management, which enables a uniform usage of all devices, thus reducing the accesses of single devices.

Thirdly, the probability distribution should not change strongly or drift upon cycling. A change of the switching probability would lead to a change in the accuracy of the approximate computing paradigm, where an increase or a decrease of the accuracy is possible. Correcting a possible shift on-chip would require to re-analyze the switching probability after a certain number of cycles and storing the determined voltage levels in a look-up table. If the shift is deterministic, the shift may be compensated by including a predetermined sliding voltage over cycling time, which, of course, requires additional circuitry.

Another aspect is the device-to-device variability. The operating voltages will be defined based on the complete array statistics. This will most probably lead to a deviation in the switching probability for individual cells. It is an open question how this device-to-device variability will influence the total accuracy of a function as many cells are involved in an arithmetic operation. In general, the device-to-device variability should be as small as possible, which is anyway the requirement for the application of memristive devices as memory cells.

**Figure 5:**
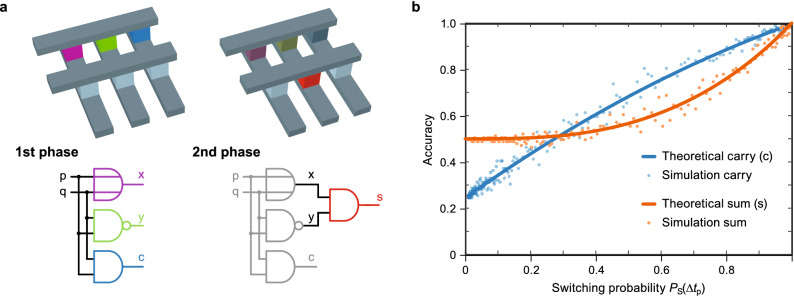
(**a**) The process of the arithmetic operation within the crossbar with the two phases to produce the sum and carry bits. (**b**) The theoretical and simulated outputs for the adder circuit.

One of the main considerations to use approximate computing is to trade the precision against energy and/or time. The time savings are obvious if the cycle time is reduced on the cost of the precision (cf. Fig. S2). In this case, the pulse length is varied while keeping the voltage constant. Increasing the pulse lengths leads to an increase in the switching probability as has been also demonstrated in literature^30,38^. Thus, at higher speed the switching probability and, in turn, the accuracy will decrease. In this case, there is a tradeoff between accuracy and switching speed. The switching energy savings is more complex, since the energy consumption of the array consists of three components. The first is the switching energy of the device ($E_{\text {switching}}$) , the second is the charging energy of the array($E_{\text {charging}}$) and the third is the energy consumed by non-operating cells ($E_{\text {leakage}}$). We consider the worst case (wc), in which the switching device is in the LRS for the duration of a pulse and the switching energy is calculated by $E_{\text {switching}}=V^2/R_{\text {LRS}}\cdot \Delta t_{\text {p}}$. Here, the device would be set with a 100% probability for $V=0.76\,\text {V}$, whereas $V=0.7\,\text {V}$ results in a 70% switching probability. Thus, by reducing the switching probability by 30% the voltage is reduced by 8% and thus the energy reduction is 15%. The charging energy can be estimated by $E_{\text {charging}}=V^2\cdot C$ and the leakage energy is approximated by $E_{\text {leakage}}= V^2/R\cdot \Delta t_{\text {p}}$. As the voltage dependence of all three cases is the same, the energy savings follow the same trend. Thus, by lowering the voltage by 10% the energy of all three components is reduced by 19% in a first approximation.

Furthermore, the outcome of cascaded gates is of interest since cascaded gates are required for more complex functions e.g. an arithmetic operator. A new probabilistic dimension is added into the analysis and requires the inclusion of further parameters, since now some inputs are not deterministic anymore. For incorporating the stochasticity of BRS-based gates into an arithmetic operator, the basic building block is a half adder. It is composed of an XOR gate for calculating the sum bit, and an AND gate for calculating the carry bit. Figure 5a shows the two phases of operation within the crossbar for attaining the sum and carry bits of the addition. The logic equations for the output bits $S_\text {out}$ and $C_\text {out}$ are

8 $$\begin{aligned} \begin{aligned} S_\text {out}&= \text {p XOR q} \\ C_\text {out}&= \text {p AND q} . \end{aligned} \end{aligned}$$

In CRS-logic, however, only 14 out of the 16 logic gates operators can be implemented with a single BRS device. The remaining gates of XOR and XNOR can be attained by a combination of the basic gates. For example, XOR is obtained by applying the inputs (p, q) to one OR gate and one NAND gate in parallel. The resulting outputs serve as inputs for an AND gate building a two-stage cascade. The equation of the operation is

9 $$\begin{aligned} \text {p XOR q} = (\text {p OR q})~~\text {AND}~~(\text {p NAND q}). \end{aligned}$$

In order to analyze the behavior of this XOR gate, the assumption of having deterministic inputs does not hold valid anymore. Especially, at the input of the AND gate used to calculate the sum. Hence, in addition to the analysis of the AND gate, a distinct set of output probabilities is formulated along with the accuracy of the sum and carry bit. Tracing back the probabilities for each input combination, the output probabilities for the AND gate with probabilistic inputs are

10 $$\begin{aligned} P_\text {out,AND}\text {(`0')} = P_\text {s}(\Delta t_\text {p})^2 \end{aligned}$$

and

11 $$\begin{aligned} P_\text {out,AND}\text {(`1')} = \frac{1}{2}(2 - 2P^2_\text {s}(\Delta t_\text {p}) + 2P^3_\text {s}(\Delta t_\text {p}) + P^4_\text {s}(\Delta t_\text {p}) - 2P^5_\text {s}(\Delta t_\text {p}) + P^6_\text {s}(\Delta t_\text {p})) \end{aligned}$$

The sum bit $S_\text {out}$ will have two entries for ‘0’  and two entries for ‘1’. Thereby, the accuracy of the sum bit is specified as

12 $$\begin{aligned} \text {Accuracy}_\text {XOR} = \text {Accuracy}_{S_\text {out}} = \frac{1}{4}(2 + 2P^3_\text {s}(\Delta t_\text {p}) + P^4_\text {s}(\Delta t_\text {p}) - 2P^5_\text {s}(\Delta t_\text {p}) + P^6_\text {s}(\Delta t_\text {p})) \end{aligned}$$

The derivation of (10)-(12) is given in the supplementary information. Since the carry bit $C_\text {out}$ is calculated by an AND gate, the accuracy is equal to that of the deterministic input. Thus, the accuracy only depends on the switching probability $P_\text {s}(\Delta t_\text {p})$ as its inputs are deterministic:

13 $$\begin{aligned} \text {Accuracy}_\text {AND} = \text {Accuracy}_{C_\text {out}} = \frac{1}{4}(1 + 4P_\text {s}(\Delta t_\text {p}) - P_\text {s}^2(\Delta t_\text {p})). \end{aligned}$$

The theoretical analysis was also verified with simulation of the gates based on the stochastic model outlined in the methods section. Figure 5b shows the accuracy for the sum and carry bit predicted by theory and simulation. Here, the performance of the sum bit starts off at 0.5, as in two cases the output should be ‘1’  and $\hbox {P}_\text {out}$(‘1’) = 1 holds for an AND gate. The accuracy of the carry bit starts with a lower performance, but catches up at a switching probability of 0.3, and has a better performance onward.

In summary, we showed an experimental proof-of-concept of approximate computing using bipolar resistive switching devices. The intrinsic stochasticity of the switching process in BRS devices can be used to tune the accuracy of CRS-logic gates. This offers a design space for the energy and delay required for a particular application at hand. Depending on the applied time period, nearly a 100% accuracy of operation is achievable at a lower nominal voltage at the expense of longer delay. Fixed accuracy levels for a range of voltages and time allow for diverse options in the design space allocation. In general, edge computing applications, such as in-sensor processing^39^ are potential candidates that could benefit from the operation of these devices. The area and energy efficiency are put forward in compromise to the latency that is relaxed in the normal operation of sensors. In each sensing cycle, the devices have ample time to process the data locally. Hence, addressing the privacy and communication concerns by sending processed data or important results rather than congesting the communication channel for cloud processing. Based on the measurement results obtained from ECM and VCM devices, device requirements for this application have been deduced. The most important one is that the switching process should depend as little as possible on the former device state to keep a constant switching probability. As ECM and VCM devices offer the required stochasticity, approximate computing approaches should be also feasible using other memristive logic approaches than CRS-logic.

## Methods

### Measurement setup

A measurement setup of a Keithley 4200 and two Keithley 4225 PMUs with associated remote amplifiers were used to perform the pulse measurements. To perform the measurement the two device terminals $T_1$ and $T_2$ are connected by probing needles to the Keithley measurement system. All experimental data shown for the VCM cell is collected on one device.

### Fabrication

The fabrication procedure for the $\hbox {ZrO}_\text {x}$-based VCM device stack is the following: 30 nm thick Pt bottom electrodes are prepared by direct current (DC) magnetron sputtering on an oxidized Si wafer with a 10 nm-thick $\hbox {TiO}_\text {2}$ adhesion layer, which is structured by a standard photolithography step. After a lift-off and coverage of the contacting pads with a positive lithography step, 6 nm $\hbox {ZrO}_\text {x}$ are then deposited by reactive RF magnetron sputtering from a metallic Zr target with an $\hbox {O}_\text {2}$ content of 5% in the plasma. After another lift-off and photolithography step 20 nm Ta and 30 nm Pt were RF sputtered and structured by a third lift-off process. The used cell area is 7x7 µ$\hbox {m}^\text {2}$.

For the $\hbox {SiO}_2$/CuS/Cu-based ECM stack, the lithography steps are identical. After the Pt bottom electrodes are processed and partially covered, 10 nm $\hbox {SiO}_2$ were RF sputtered. Followed by the third lithography step, 5 nm of Cu were deposited via electron beam evaporation and subsequently sulfurized at 80 $^{\circ }$C in vacuum for 10 min^40^. The stack is completed by deposition of a 30 nm thick Cu layer (e-beam evaporation) and 30 nm thick Pt layer (RF sputtered) to prevent excessive oxidation. The used cell area is 10 x 10 µ$\hbox {m}^\text {2}$.

### Simulation model

A statistical based model^2,17,41^ is used to simulate the behavior of the memristor under probabilistic operation. The model assumes two ohmic resistances of 1 k$\Omega$ (LRS) and 1 M$\Omega$. The SET (RESET) switching is assumed to occur at a threshold voltage of 2.67 V (− 2.67 V). The model introduces variability into the physical model by extracting from the experimental data the switching times at different voltages. The main concept lies in picking a sample from a uniform random distribution and comparing it to the probability of switching calculated at each point in time according to Eq. (6). In case the calculated probability is greater than the chosen sample, the threshold voltage is set to this value and the switching occurs. The model is implemented as Verilog model. The corresponding code lines are listed in^17^. The variation of the threshold voltage is linked to several processes. There is a deterministic component determining the variability and a stochastic one. The stochasticity origins from the driving ionic processes. The switching relies on the motion of ions and concurrent redox reactions. Both processes are stochastic in nature. Thus, the switching itself is stochastic^42,43^. Moreover, from cycle to cycle the filament geometry can change or the filament can even form in a different location^44^. Besides the stochastic contribution, there is also a strong state-dependence of the switching time as discussed in^38,45^, i.e. the resistance of the LRS or HRS determines the switching time. This state-dependence can be modeled using physics-based models^38,46^. Here, the state-dependence is not considered directly in the model, but manifests in the stochasticity of the threshold voltage (variability in switching time). The model is used to verify the theoretical accuracy attained with the simulation of the BRS device for 100 times for all of the possible combinations of the logic gate, and recording the output states accordingly.

## Supplementary Information

Supplementary Information.
