# Supplementary material for: Theory and experimental verification of configurable computing with stochastic memristors
Source: Sci Rep. 2021 Feb 18;11:4218. doi: 10.1038/s41598-021-83382-y (PMC7893165; doi:10.1038/s41598-021-83382-y)
Supplement: Supplementary file 1 — Supplementary Information. [file 41598_2021_83382_MOESM1_ESM.pdf]

# Supplementary Information: Theory and Experimental Verification of Configurable Computing with Stochastic Memristors

Rawan Naous[1,2,+], Anne Siemon[3,4,+], Michael Schulten[3,4], Hamazah Alahmadi[1], Andreas Kindsmüller[3,4], Michael Lübben[3,4], Arne Heitmann[4,5], Rainer Waser[3,4,5,6], Kaled Nabil Salama[1] and Stephan Menzel[4,6,*]

[2] Department of Electrical Engineering and Computer Science, University of California Berkeley, Berkeley, CA, USA

[1] Computer, Electrical and Mathematical Sciences and Engineering Division, King Abdullah University of Science and Technology, Thuwal, Saudi Arabia

[3] Institut für Werkstoffe der Elektrotechnik II (IWE II), RWTH Aachen University, Aachen, Germany

[4] JARA - Fundamentals for Future Information Technology, Jülich, Germany

[5] Peter Grünberg Institut 10 (PGI-10), Forschungszentrum Jülich GmbH, Jülich, Germany

[6] Peter Grünberg Institut 7 (PGI-7), Forschungszentrum Jülich GmbH, Jülich, Germany

[*] Corresponding Author: st.menzel@fz-juelich.de

[+] These authors contributed equally to this work

## Probabilistic Switching of VCM and ECM Cells

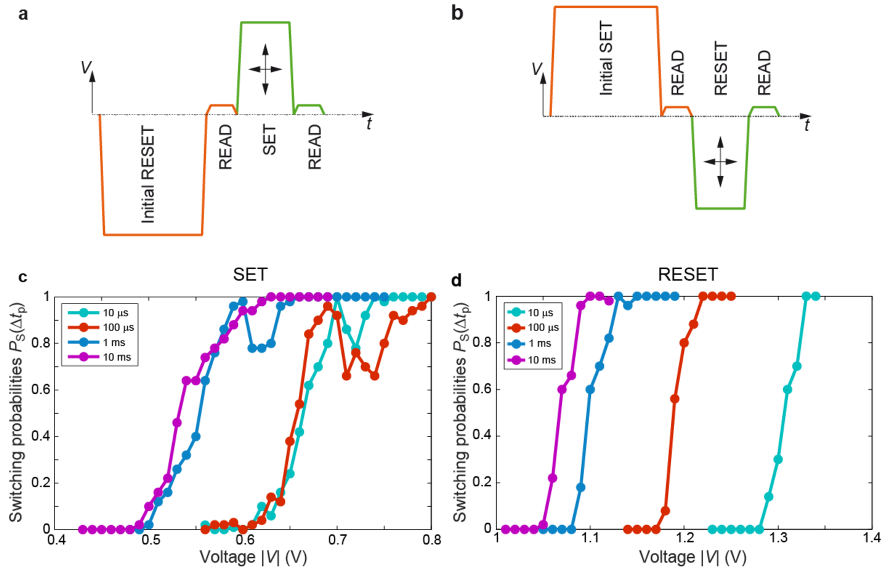


Figure S1: VCM measurements without refresh cycles for different pulse lengths and heights. Pulse schemes for a) SET and b) RESET probabilities. First, an initial 1 ms (4 ms) long rectangular RESET (SET) of -1.4 V (1 V) is applied, which is followed by 0.1 V, 1 ms long READ pulse. The succeeding SET (RESET) pulse has variable amplitude and length. Finally, a 0.1 V, 1 ms long READ pulse is applied.

Success rates of 50 repetitions for each point for c) SET and d) RESET.

In Figure S1, the results of the measurements without sticking prevention are shown. It should be noticed, that the wanted dependency is mostly matched. Thus, a reduction in time while keeping the voltage constant results in a lower success rate. The same behavior is shown for a reduction of the voltage while keeping the timing constant. In the SET success rate are some discrepancies of the predicted behavior in the higher success rates. These could be due to the relatively small amount of repetitions or due to the high variability of the devices. In the measurements, it

was observed that the initial resistance state showed a strong history dependence of the applied pulses. Thus, we introduced the unsticking preventions as shown in the main manuscript. For the success rate measurements, the difference in the two procedures appears at high voltages. In the case without unsticking preventions, a dip in the SET success rates is observed in the high voltage regime.

Figure S2 shows the probability of the switching for the SET and RESET process of the VCM cell at different voltages. It is obvious that a reduction of the pulse length would lead to a lower switching probability.

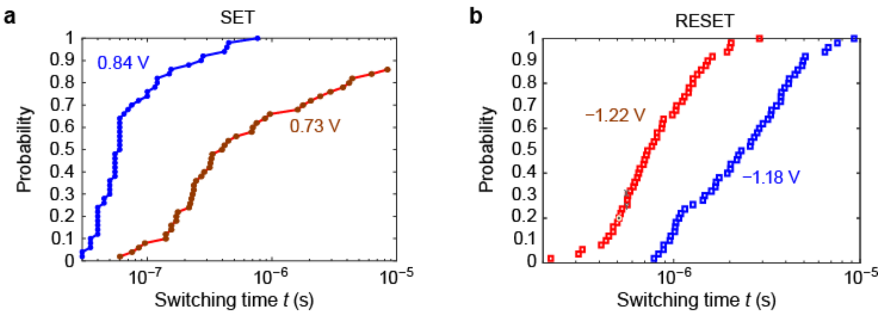


Figure S2: Switching probability of the VCM cell for (a) SET and (b) RESET as a function of time.

Similar to the VCM cells, the switching kinetics of the ECM cells were investigated. The switching time was evaluated for all of the 50 cycles that switched successfully. The switching times are extracted in a similar way as for the VCM cells (see below). For the SET operation, the critical current level corresponds to a resistance of $R_{\text{crit}} = 1.5\,\text{k}\Omega$. For extracting the RESET time, a critical current level of $I_{\text{crit,RESET}} = -140\,\mu\text{A}$ at $0.22\,V$ was chosen. Then, the absolute value of the critical current is reduced by $5\,\mu\text{A}$ per $10\,\text{mV}$ voltage increment (e.g. $-130\,\mu\text{A}$ at $-0.2\,\text{V}$).

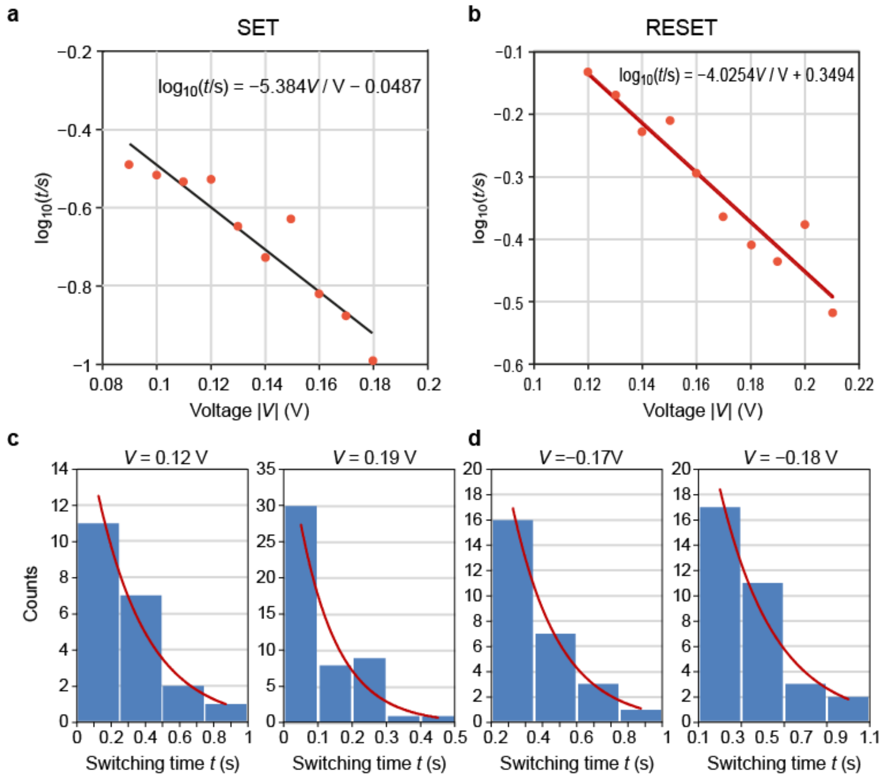


Figure S3: The time-voltage relationship for the ECM device during the (a) SET operation and (b) RESET operation. The experimental fitting for the switching times at different voltages to the exponential distribution, leading to a Poisson process for the (c) SET and (d) RESET switching events.

The resulting switching time obeys an exponential relation on the applied voltage as shown for the VCM cells in Figure S3(a) and (b). The fitting equation are given in the figures. In Figure S3(c),(d), it is demonstrated that the SET/RESET switching times can be fitted using a Poisson distribution.

In Figure S4, the results of the ECM measurements are shown. As expected, the wanted dependency is mostly matched. A reduction of the voltage while keeping the timing constant

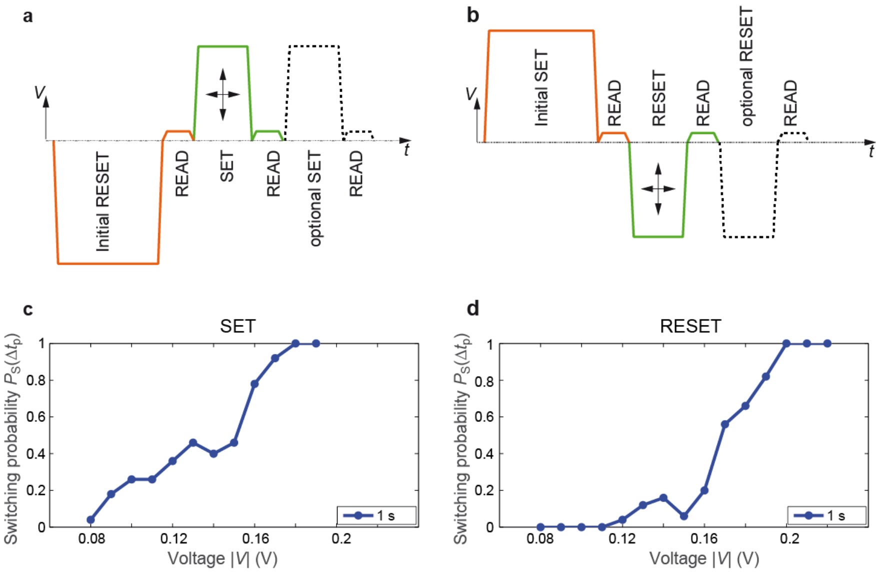


Figure S4: ECM measurements with refresh cycles for different pulse heights. Pulse schemes for a) SET and b) RESET probabilities. First, an initial 1 s long rectangular RESET (SET) of -0.2 V (0.5 V) is applied, which is followed by 0.1 V, 1 ms long READ pulse. The succeeding SET (RESET) pulse has variable amplitude and length. Finally, a 0.1 V, 1 ms long READ pulse is applied. If the device did not switch and optional 1 s long SET (RESET) pulse of 0.5/,V (-0.2 V) is applied. Success rates of 50 repetitions for each point for c) SET and d) RESET.

shows a lower success rate. The sticking problem was also present in the ECM devices since no current compliance or other protection mechanisms were used. Here the unsticking cycles were performed as non-triangular pulses and there is no additional cycling before the tested pulse.

The switching times (as shown in Fig. 3 of the main manuscript) for the VCM cells are extracted from the *I*-*t* traces recorded during the programming pulses. Figure S5 shows the transient currents for the SET and the RESET process for all cycles for a specific voltage. For both processes, a clear transition from one state to the other appears. The crossing point of the dashed lines

with the $I$-$t$ traces determines the switching time. For the SET operation, the critical current level corresponds to a resistance of $R_{\text{crit}} = 1.5\,\text{k}\Omega$, i.e. $I_{\text{crit,SET}} = V_{\text{pulse}}/R_{\text{crit}}$. This is the same condition as for defining the success rate from the Read pulse. The HRS, however, shows a nonlinear $I - V$ characteristics, thus the large signal resistance at the pulse voltage will be lower than the read resistance. In addition, the RESET transition is more gradual in general. Thus, a different criterion is chosen. Starting with a critical current level of $I_{\text{crit,RESET}} = -1.25\,\text{mA}$ at -1.23 V, the absolute value of the critical current is reduced by $50\,\mu$A per $10\,$mV voltage increment (e.g. -1.2 mA at -1.22 V).

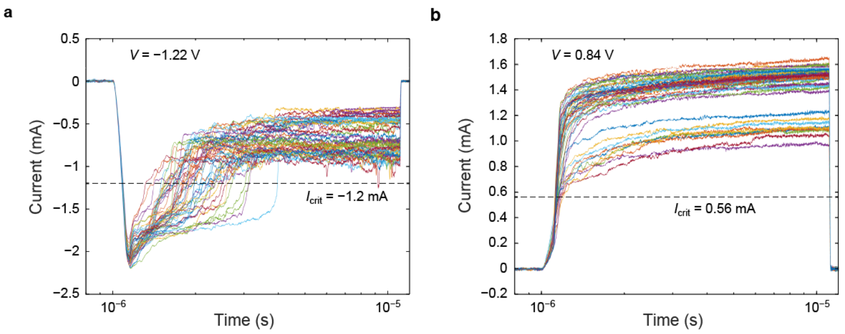


Figure S5: Exemplary $I$-$t$ traces recorded during the programming of the VCM cell for the (a) SET operation and (b) RESET operation. The amplitude of the SET pulse is 0.84 V and the amplitude of the RESET pulse -1.22 V. The dashed line shows the criterion for determining the switching time used for the fits in Fig. 3 of the main manuscript.

## Measurement of the NAND Gate

In Figure S6, the results of the NAND gate measurements with unsticking cycles for all input combinations are depicted. The trends of the input combinations (p = ‘0’; q = ‘0’), (p = ‘1’; q =

‘0’) and (p = ‘1’; q = ‘1’) of the theory are also visible in the measurements. Only the measurements of (p = ‘0’; q = ‘1’) have a wrong trend. Here, the RESET of the second cycle is weaker (shorter pulse length and lower pulse height) than the initialization pulse of the SET measurements. Thus, the device will be in a lower ohmic HRS state than in the SET measurements and it will switch more easily back to the LRS state.

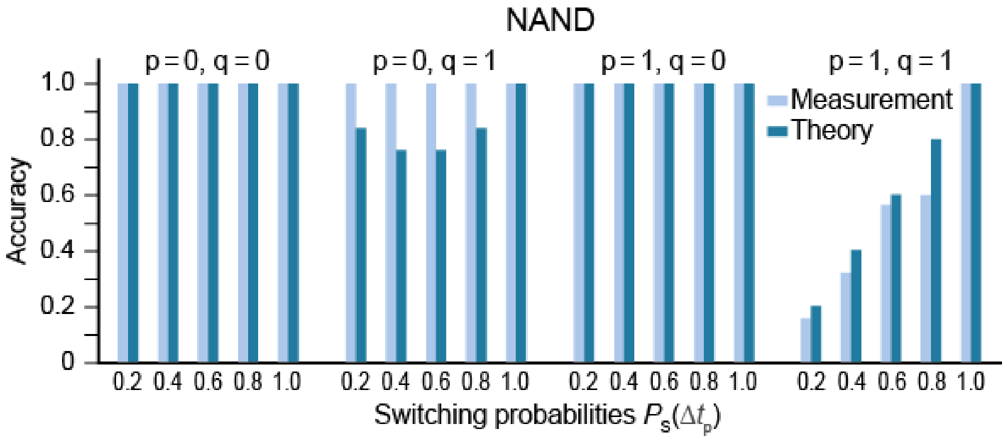


Figure S6: Comparison of the theoretical and measurement results of the NAND gate for the 0.2, 0.4, 0.6, 0.8 and 1.0 switching probabilities points of the measurements for all four input combinations.

**Probabilistic Analysis of CRS Logic Gates**

In Figure S7, the probabilistic analysis for the logic gates in terms of the output probabilities and the corresponding accuracy is depicted. The gates form the basis for any logic operation and could be cascaded to have further arithmetic operations as well. 100 simulation runs for each gate are conducted, and the resulting output is noted in regard to the expected output behavior for all the

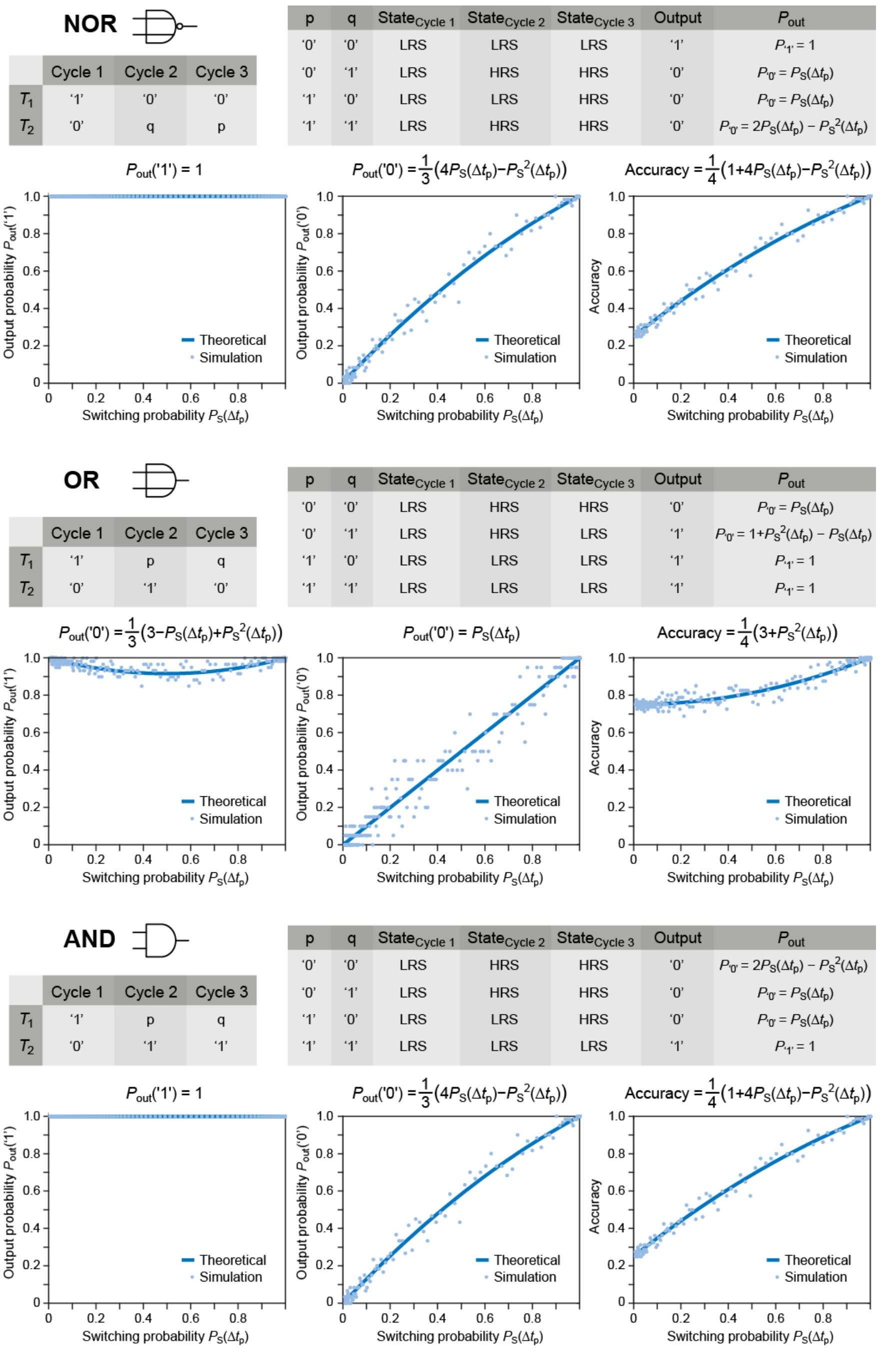


Figure S7: The probabilistic analysis of logic gates with theoretical and simulation-based on behavioral modeling of stochastic devices.

entries in the truth table. The simulations showed a high level of matching with the analytical equations.

Figure S8 and S9 show the probabilistic analysis for the logic gates True, p, and q (Figure S8) and IMP, RIMP (Figure S9). As defined in the main manuscript the first initialization cycle is assumed to be deterministic. For the gates p, q, IMP and RIMP, the first cycle is identical to the True gate. Thus, the True gate is assumed to be deterministic to be consistent throughout the manuscript.

Figure S10 and S11 show the probabilistic analysis for the logic gates False, not p, and not q (Figure S10) and NIMP, RNIMP (Figure S11). As defined in the main manuscript the first initialization cycle is assumed to be deterministic. For the gates not p, not q, NIMP and RNIMP, the first cycle is identical to the False gate. Thus, the False gate is assumed to be deterministic to be consistent throughout the manuscript.

**Derivation of the Accuracy of an XOR Gate**

In order to derive the output probabilities of an XOR gate and its accuracy, it needs to be considered that the inputs of the second phase AND are non-deterministic anymore. They rather depend on the output probabilities of the NAND and the OR gate in the first phase of the XOR operation. It also needs to be considered that the output after the first phase is incorrect, but the final output after the second state can be correct. Thus, every case needs to be analyzed separately.

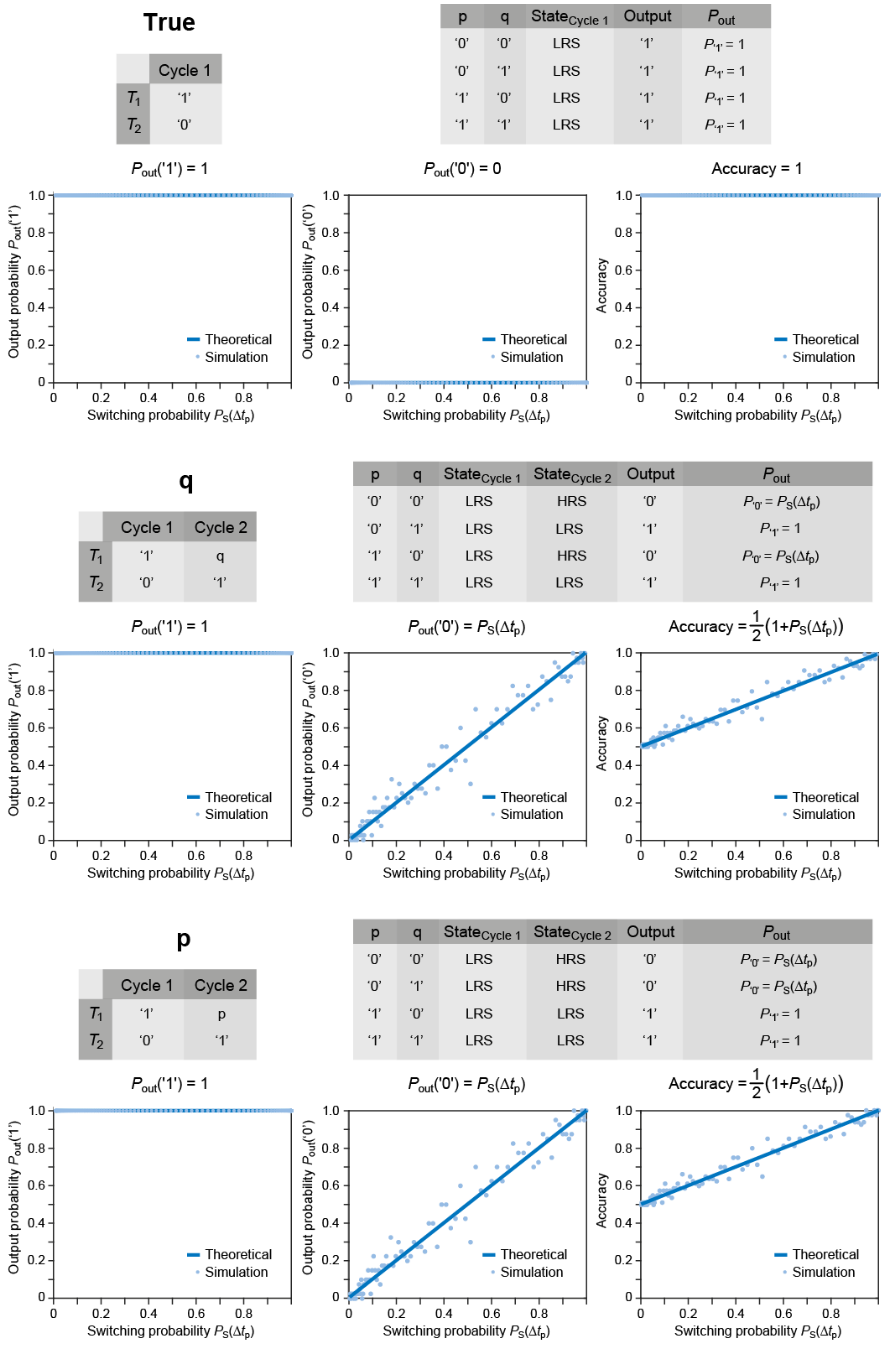


Figure S8: The probabilistic analysis of logic gates with theoretical and simulation-based on behavioral modeling of stochastic devices.

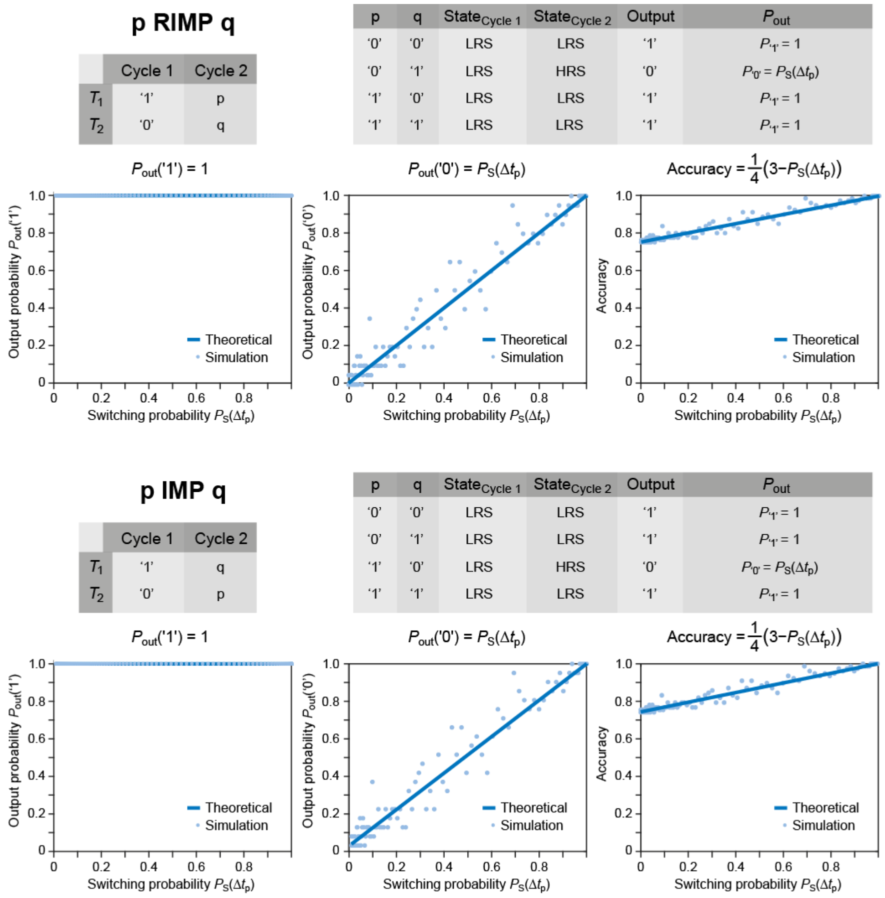


Figure S9: The probabilistic analysis of logic gates with theoretical and simulation-based on behavioral modeling of stochastic devices.

**False**

| | Cycle 1 |
|---|---|
| $T_1$ | '0' |
| $T_2$ | '1' |

| p | q | $State_{Cycle\ 1}$ | Output | $P_{out}$ |
|---|---|---|---|---|
| '0' | '0' | HRS | '0' | $P_{'1'} = 1$ |
| '0' | '1' | HRS | '0' | $P_{'1'} = 1$ |
| '1' | '0' | HRS | '0' | $P_{'1'} = 1$ |
| '1' | '1' | HRS | '0' | $P_{'1'} = 1$ |

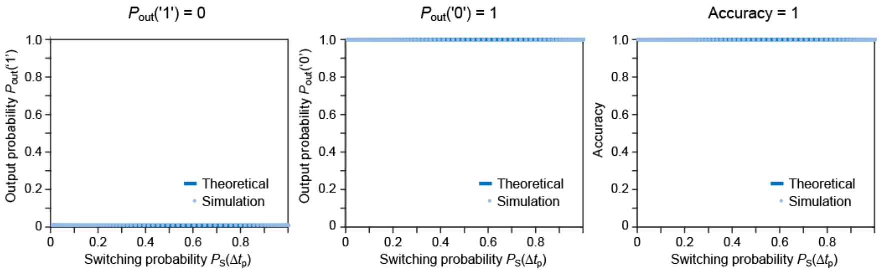


**not q**

| | Cycle 1 | Cycle 2 |
|---|---|---|
| $T_1$ | '0' | '1' |
| $T_2$ | '1' | q |

| p | q | $State_{Cycle\ 1}$ | $State_{Cycle\ 2}$ | Output | $P_{out}$ |
|---|---|---|---|---|---|
| '0' | '0' | HRS | LRS | '1' | $P_{'1'} = P_S(\Delta t_p)$ |
| '0' | '1' | HRS | HRS | '0' | $P_{'0'} = 1$ |
| '1' | '0' | HRS | LRS | '1' | $P_{'1'} = P_S(\Delta t_p)$ |
| '1' | '1' | HRS | HRS | '0' | $P_{'0'} = 1$ |

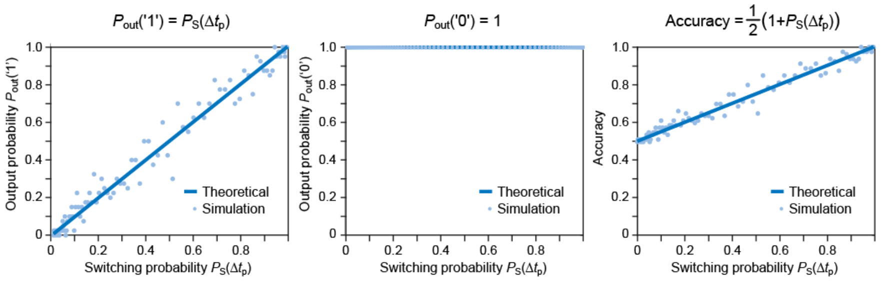


**not p**

| | Cycle 1 | Cycle 2 |
|---|---|---|
| $T_1$ | '0' | '1' |
| $T_2$ | '1' | p |

| p | q | $State_{Cycle\ 1}$ | $State_{Cycle\ 2}$ | Output | $P_{out}$ |
|---|---|---|---|---|---|
| '0' | '0' | HRS | LRS | '1' | $P_{'1'} = P_S(\Delta t_p)$ |
| '0' | '1' | HRS | LRS | '1' | $P_{'1'} = P_S(\Delta t_p)$ |
| '1' | '0' | HRS | HRS | '0' | $P_{'0'} = 1$ |
| '1' | '1' | HRS | HRS | '0' | $P_{'0'} = 1$ |

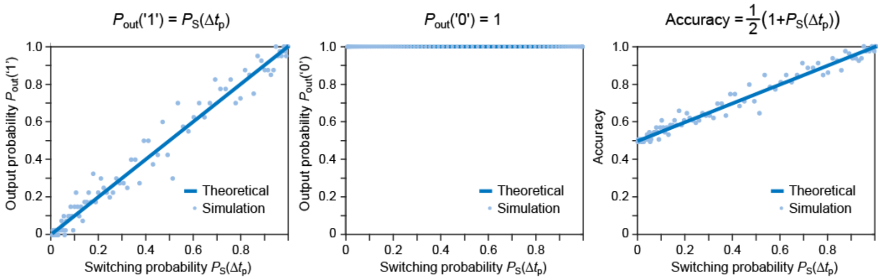


Figure S10: The probabilistic analysis of logic gates with theoretical and simulation-based on behavioral modeling of stochastic devices.

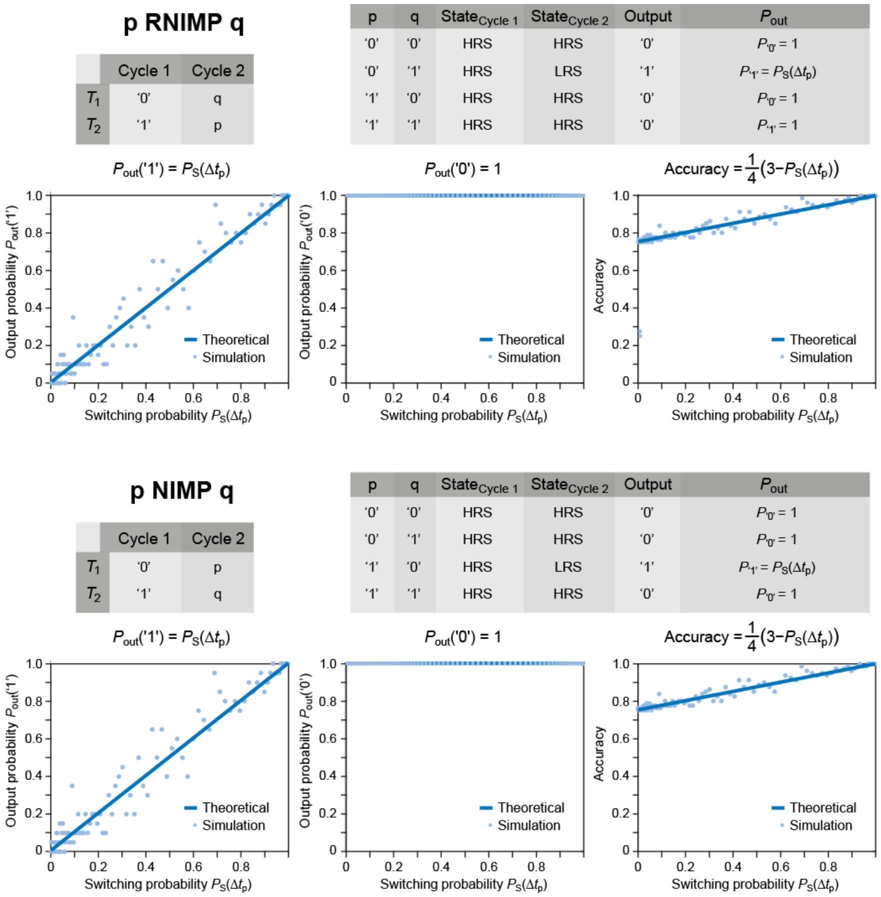


Figure S11: The probabilistic analysis of logic gates with theoretical and simulation-based on behavioral modeling of stochastic devices.

First, we always need to derive the probabilities of the outputs of the first gate stage, i.e. $P_{\text{out,NAND (p,q)}}(\text{‘1’})$, $P_{\text{out,NAND (p,q)}}(\text{‘0’})$, $P_{\text{out,OR (p,q)}}(\text{‘1’})$ and $P_{\text{out,OR (p,q)}}(\text{‘0’})$. From this we can calculate the probability of the input combinations of the second stage, the AND gate, $P_{\text{in,AND (p.q)}}(\text{x,y})$, where x and y denote the outputs of the NAND and the OR gate, respectively. These probabilities are multiplied with the probabilities of the AND gate to achieve the correct result of the XOR gate for the inputs p and q.

For the ease of readability $P_{\text{S}}$ is used instead of $P_{\text{S}}(t)$ in the following derivation.

For the inputs p = ‘0’ and q = ‘0’ of the XOR gate, the input probabilities of the AND gates are

$$P_{\text{in,AND (‘0’,‘0’)}}(\text{‘0’,‘0’}) = P_{\text{out,NAND,(‘0’,‘0’)}}(\text{‘0’}) * P_{\text{out,OR (‘0’,‘0’)}}(\text{‘0’}) = 0 * P_{\text{S}} = 0, \tag{1}$$

$$P_{\text{in,AND (‘0’,‘0’)}}(\text{‘0’,‘1’}) = P_{\text{out,NAND (‘0’,‘0’)}}(\text{‘0’}) * P_{\text{out,OR (‘0’,‘0’)}}(\text{‘1’}) = 0 * (1 - P_{\text{S}}) = 0, \tag{2}$$

$$P_{\text{in,AND (‘0’,0’)}}(\text{‘1’,‘0’}) = P_{\text{out,NAND (‘0’,‘0’)}}(\text{‘1’}) * P_{\text{out,OR (‘0’,‘0’)}}(\text{‘0’}) = 1 * P_{\text{S}}, \tag{3}$$

and

$$P_{\text{in,AND (‘0’,‘0’)}}(\text{‘1’,‘1’}) = P_{\text{out,NAND (‘0’,‘0’)}}(\text{‘1’}) * P_{\text{out,OR (‘0’,‘0’)}}(\text{‘1’}) = 1 * (1 - P_{\text{S}}). \tag{4}$$

The probability of the correct output of the XOR gate is

$$\begin{aligned}P_{\text{out,XOR (‘0’,‘0’)}}(\text{‘0’}) &= P_{\text{in,AND (‘0’,‘0’)}}(\text{‘0’},\text{‘0’}) * P_{\text{out,AND (‘0’,‘0’)}}(\text{‘0’}) \\ &+ P_{\text{in,AND (‘0’,‘0’)}}(\text{‘0’},\text{‘1’}) * P_{\text{out,AND (‘0’,‘1’)}}(\text{‘0’}) \\ &+ P_{\text{in,AND (‘0’,‘0’)}}(\text{‘1’},\text{‘0’}) * P_{\text{out,AND (‘1’,‘0’)}}(\text{‘0’}) \\ &+ P_{\text{in,AND (‘0’,‘0’)}}(\text{‘1’},\text{‘1’}) * P_{\text{out,AND (‘1’,‘1’)}}(\text{‘0’}) \\ &= 0 * (2P_{\text{S}} - P_{\text{S}}^2) + 0 * P_{\text{S}} + P_{\text{S}} * P_{\text{S}} + (1 - P_{\text{S}}) * 0 \\ &= P_{\text{S}}^2.\end{aligned} \tag{5}$$

Thus, if $P_{\text{S}}(t)$ is zero, the result is never correct.

For the inputs p = ‘0’and q = ‘1’of the XOR gate, the input probabilities of the AND gates are

$$\begin{aligned}P_{\text{in,AND (‘0’,‘1’)}}(\text{‘0’},\text{‘0’}) &= P_{\text{out,NAND (‘0’,‘1’)}}(\text{‘0’}) * P_{\text{out,OR (‘0’,‘1’)}}(\text{‘0’}) \\ &= (P_{\text{S}} - P_{\text{S}}^2) * (P_{\text{S}} - P_{\text{S}}^2) = P_{\text{S}}^2 - 2P_{\text{S}}^3 + P_{\text{S}}^4,\end{aligned} \tag{6}$$

$$\begin{aligned}P_{\text{in,AND (‘0’,‘1’)}}(\text{‘0’},\text{‘1’}) &= P_{\text{out,NAND (‘0’,‘1’)}}(\text{‘0’}) * P_{\text{out,OR (‘0’,‘1’)}}(\text{‘1’}) \\ &= (P_{\text{S}} - P_{\text{S}}^2) * (1 + P_{\text{S}}^2 - P_{\text{S}}) = P_{\text{S}} - 2P_{\text{S}}^2 + 2P_{\text{S}}^3 - P_{\text{S}}^4,\end{aligned} \tag{7}$$

$$\begin{aligned}P_{\text{in,AND (‘0’,‘1’)}}(\text{‘1’},\text{‘0’}) &= P_{\text{out,NAND (‘0’,‘1’)}}(\text{‘1’}) * P_{\text{out,OR (‘0’,‘1’)}}(\text{‘0’}) \\ &= (1 + P_{\text{S}}^2 - P_{\text{S}}) * (P_{\text{S}} - P_{\text{S}}^2) = P_{\text{S}} - 2P_{\text{S}}^2 + 2P_{\text{S}}^3 - P_{\text{S}}^4,\end{aligned} \tag{8}$$

and

$$P_{\text{in,AND ('0','1')}}(\text{'1','1'}) = P_{\text{out,NAND ('0','1')}}(\text{'1'}) * P_{\text{out,OR ('0','1')}}(\text{'1'})$$
$$= (1 + P_{\text{S}}^2 - P_{\text{S}}) * (1 + P_{\text{S}}^2 - P_{\text{S}}) \tag{9}$$
$$= 1 - 2P_{\text{S}} + 3P_{\text{S}}^2 - 2P_{\text{S}}^3 + P_{\text{S}}^4.$$

The probability of the correct output of the XOR gate is

$$P_{\text{out,XOR ('0','1')}}(\text{'1'}) = P_{\text{in,AND ('0','1')}}(\text{'0','0'}) * P_{\text{out,AND ('0','0')}}(\text{'1'})$$
$$+ P_{\text{in,AND ('0','1')}}(\text{'0','1'}) * P_{\text{out,AND ('0','1')}}(\text{'1'})$$
$$+ P_{\text{in,AND ('0','1')}}(\text{'1','0'}) * P_{\text{out,AND ('1','0')}}(\text{'1'})$$
$$+ P_{\text{in,AND ('0','1')}}(\text{'1','1'}) * P_{\text{out,AND ('1','1')}}(\text{'1'})$$
$$= (P_{\text{S}}^2 - 2P_{\text{S}}^3 + P_{\text{S}}^4) * (1 + P_{\text{S}}^2 - 2P_{\text{S}}) \tag{10}$$
$$+ (P_{\text{S}} - 2P_{\text{S}}^2 + 2P_{\text{S}}^3 - P_{\text{S}}^4) * (1 - P_{\text{s}})$$
$$+ (P_{\text{S}} - 2P_{\text{S}}^2 + 2P_{\text{S}}^3 - P_{\text{S}}^4) * (1 - P_{\text{s}})$$
$$+ (1 - 2P_{\text{S}} + 3P_{\text{S}}^2 - 2P_{\text{S}}^3 + P_{\text{S}}^4) * 1$$
$$= 1 - 2P_{\text{S}}^2 + 2P_{\text{S}}^3 + P_{\text{S}}^4 - 2P_{\text{S}}^5 + P_{\text{S}}^6.$$

For this case, the probability of getting the correct result is always higher than 0.7.

For the inputs p = '1'and q = '0'of the XOR gate, the input probabilities of the AND gates are

$$P_{\text{in,AND ('1','0')}}(\text{'0','0'}) = P_{\text{out,NAND ('1','0')}}(\text{'0'}) * P_{\text{out,OR ('1','0')}}(\text{'0'}) = 0 * 0 = 0, \tag{11}$$

$$P_{\text{in,AND ('1','0')}}(\text{'0','1'}) = P_{\text{out,NAND ('1','0')}}(\text{'0'}) * P_{\text{out,OR ('1','0')}}(\text{'1'}) = 0 * 1 = 0, \tag{12}$$

$$P_{\text{in,AND (`1',`0')}}(\text{`1',`0'}) = P_{\text{out,NAND (`1',`0')}}(\text{`1'}) * P_{\text{out,OR (`1',`0')}}(\text{`0'}) = 1 * 0 = 0, \tag{13}$$

and

$$P_{\text{in,AND (`1',`0')}}(\text{`1',`1'}) = P_{\text{out,NAND (`1',`0')}}(\text{`1'}) * P_{\text{out,OR (`1',`0')}}(\text{`1'}) = 1 * 1 = 1. \tag{14}$$

The probability of the correct output of the XOR gate is

$$\begin{aligned} P_{\text{out,XOR (`1',`0')}}(\text{`1'}) = {} & P_{\text{in,AND (`1',`0')}}(\text{`0',`0'}) * P_{\text{out,AND (`0',`0')}}(\text{`1'}) \\ & + P_{\text{in,AND (`1',`0')}}(\text{`0',`1'}) * P_{\text{out,AND (`0',`1')}}(\text{`1'}) \\ & + P_{\text{in,AND (`1',`0')}}(\text{`1',`0'}) * P_{\text{out,AND (`1',`0')}}(\text{`1'}) \\ & + P_{\text{in,AND (`1',`0')}}(\text{`',`1'}) * P_{\text{out,AND (`1',`1')}}(\text{`1'}) \\ = {} & 0 * (1 - 2P_{\text{s}} + P_{\text{s}}^2) + 0 * (1 - P_{\text{s}}) + 0 * (1 - P_{\text{s}}) + 1 * 1 \\ = {} & 1. \end{aligned} \tag{15}$$

Thus, the sum bit is always correct in this case.

For the inputs p = ‘1’and q = ‘1’of the XOR gate, the input probabilities of the AND gates are

$$P_{\text{in,AND (`1',`1')}}(\text{`0',`0'}) = P_{\text{out,NAND (`1',`1')}}(\text{`0'}) * P_{\text{out,OR (`1',`1')}}(\text{`0'}) = P_{\text{S}} * 0 = 0, \tag{16}$$

$$P_{\text{in,AND (`1',`1')}}(\text{`0',`1'}) = P_{\text{out,NAND (`1',`1')}}(\text{`0'}) * P_{\text{out,OR (`1',`1')}}(\text{`1'}) = P_{\text{S}} * 1 = P_{\text{S}}, \tag{17}$$

$$P_{\text{in,AND (`1',`1')}}(\text{`1',`0'}) = P_{\text{out,NAND (`1',`1')}}(\text{`1'}) * P_{\text{out,OR (`1',`1')}}(\text{`0'}) = (1 - P_{\text{S}}) * 0 = 0, \tag{18}$$

and

$$P_{\text{in,AND (`1',`1')}}(\text{`1',`1'}) = P_{\text{out,NAND (`1',`1')}}(\text{`1'}) * P_{\text{out,OR (`1',`1')}}(\text{`1'}) = (1 - P_{\text{S}}) * 1 = 1 - P_{\text{S}}. \tag{19}$$

The probability of the correct output of the XOR gate is

$$
\begin{aligned}
P_{\text{out,XOR (‘1’,‘1’)}}(\text{‘0’}) &= P_{\text{in,AND (‘1’,‘1’)}}(\text{‘0’},\text{‘0’}) * P_{\text{out,AND (‘0’,‘0’)}}(\text{‘0’}) \\
&\quad + P_{\text{in,AND (‘1’,‘1’)}}(\text{‘0’},\text{‘1’}) * P_{\text{out,AND (‘0’,‘1’)}}(\text{‘0’}) \\
&\quad + P_{\text{in,AND (‘1’,‘1’)}}(\text{‘1’},\text{‘0’}) * P_{\text{out,AND (‘1’,‘0’)}}(\text{‘0’}) \\
&\quad + P_{\text{in,AND (‘1’,‘1’)}}(\text{‘1’},\text{‘1’}) * P_{\text{out,AND (‘1’,‘1’)}}(\text{‘0’}) \\
&= 0 * (2P_{\text{s}} - P_{\text{s}}^2) + P_{\text{s}} * P_{\text{s}} + 0 * P_{\text{s}} + (1 - P_{\text{S}}) * 0 \\
&= P_{\text{s}}^2.
\end{aligned}
\tag{20}
$$

Thus, a high switching probability is required to generate a high probability of the correct XOR output.

The inputs p and q of the XOR gate are assumed to have the same probability. Thus, accuracy of the XOR gate is

$$
\begin{aligned}
\text{Accuracy}_{\text{XOR}} &= 1/4 * (P_{\text{out,XOR (‘0’,‘0’)}}(\text{‘0’}) + P_{\text{out,XOR (‘0’,‘1’)}}(\text{‘1’}) \\
&\quad + P_{\text{out,XOR (‘1’,‘0’)}}(\text{‘1’}) + P_{\text{out,XOR (‘1’,‘1’)}}(\text{‘0’})) \\
&= 1/4 * (2 + 2P_{\text{S}}^3 + P_{\text{S}}^4 - 2P_{\text{S}}^5 + P_{\text{S}}^6).
\end{aligned}
\tag{21}
$$

Likewise the probability of a correct output ‘0’is given by

$$
\begin{aligned}
P_{\text{out,XOR}}(\text{‘0’}) &= 1/2 * (P_{\text{out,XOR (‘0’,‘0’)}}(\text{‘0’}) + P_{\text{out,XOR (‘1’,‘1’)}}(\text{‘0’})) \\
&= 1/2 * (P_{\text{S}}^2 + P_{\text{S}}^2) = P_{\text{S}}^2
\end{aligned}
\tag{22}
$$

and for a correct output ‘1’it is

$$P_{\text{out,XOR}}(\text{‘1’}) = 1/2 * (P_{\text{out,XOR (‘0’,‘1’)}}(\text{‘1’}) + P_{\text{out,XOR (‘1’,‘0’)}}(\text{‘1’}))$$

$$= 1/2 * (1 + 1 - 2P_{\text{S}}^2 + 2P_{\text{S}}^3 + P_{\text{S}}^4 - 2P_{\text{S}}^5 + P_{\text{S}}^6) \qquad (23)$$

$$= 1/2 * (2 - 2P_{\text{S}}^2 + 2P_{\text{S}}^3 + P_{\text{S}}^4 - 2P_{\text{S}}^5 + P_{\text{S}}^6).$$
